# AIB1 sequestration by androgen receptor inhibits estrogen-dependent cyclin D1 expression in breast cancer cells

**DOI:** 10.1186/s12885-019-6262-4

**Published:** 2019-11-04

**Authors:** Francesca De Amicis, Chiara Chiodo, Catia Morelli, Ivan Casaburi, Stefania Marsico, Rosalinda Bruno, Diego Sisci, Sebastiano Andò, Marilena Lanzino

**Affiliations:** Department of Pharmacy, Health and Nutritional Sciences, University of Calabria, CS, 87036 Arcavacata di Rende, Italy

**Keywords:** Breast cancer, MCF-7, Androgen receptor, Estrogen receptor, Dihydrotestosterone, Estradiol, Coactivators, AIB1, SRC3, Cyclin D1

## Abstract

**Background:**

Androgens, through their own receptor, play a protective role on breast tumor development and progression and counterbalance estrogen-dependent growth stimuli which are intimately linked to breast carcinogenesis.

**Methods:**

Cell counting by trypan blu exclusion was used to study androgen effect on estrogen-dependent breast tumor growth. Quantitative Real Time RT–PCR, western blotting, transient transfection, protein immunoprecipitation and chromatin immunoprecipitation assays were carried out to investigate how androgen treatment and/or androgen receptor overexpression influences the functional interaction between the steroid receptor coactivator AIB1 and the estrogen- or androgen receptor which, in turn affects the estrogen-induced cyclin D1 gene expression in MCF-7 breast cancer cells. Data were analyzed by ANOVA.

**Results:**

Here we demonstrated, in estrogen receptor α (ERα)-positive breast cancer cells, an androgen-dependent mechanism through which ligand-activated androgen receptor (AR) decreases estradiol-induced cyclin D1 protein, mRNA and gene promoter activity. These effects involve the competition between AR and ERα for the interaction with the steroid receptor coactivator AIB1, a limiting factor in the functional coupling of the ERα with the cyclin D1 promoter. Indeed, AIB1 overexpression is able to reverse the down-regulatory effects exerted by AR on ERα-mediated induction of cyclin D1 promoter activity. Co-immunoprecipitation studies indicated that the preferential interaction of AIB1 with ERα or AR depends on the intracellular expression levels of the two steroid receptors. In addition, ChIP analysis evidenced that androgen administration decreased E_2_-induced recruitment of AIB1 on the AP-1 site containing region of the cyclin D1 gene promoter.

**Conclusions:**

Taken together all these data support the hypothesis that AIB1 sequestration by AR may be an effective mechanism to explain the reduction of estrogen-induced cyclin D1 gene activity. In estrogen-dependent breast cancer cell proliferation, these findings reinforce the possibility that targeting AR signalling may potentiate the effectiveness of anti-estrogen adjuvant therapies.

## Background

The high frequency of androgen receptor (AR) expression in primary breast tumours (70–90%) [1, 2] suggests that androgens are important modulators of breast cancer cell proliferation [3]. Androgens have inhibitory effects on the proliferation of normal breast epithelial cells and play a protective role in the pathogenesis of breast cancer growth [4, 5]. Indeed, several events involved in breast cancer genesis or progression have been shown to alter AR expression or function. In BRCA1-mutated tumours, loss of AR expression, and thus loss of AR signalling, supports neoplastic transformation of mammary epithelial cells [6]. AR signalling influence on breast cancer depends on the contest of the different subtype of disease [3] as indicated by a number of studies showing that the expression of AR is a favourable determinant of survival in estrogen receptor alpha (ERα)-positive, but not ERα-negative breast cancer patients [7–9]. These observations have been recently confirmed. A wide study, conducted on 4147 pre- and postmenopausal women with invasive breast cancer found that, in the 7 years following diagnosis, AR expression was associated with improved prognosis in ERα-positive tumors and worse prognosis in ERα-negative ones [10].

Similarly, in luminal ERα-positive breast cancer cell lines, AR exerts an anti-proliferative effect [11–16] while in MDA-MB-453, a model of ERα-negative apocrine disease, activated AR can induce cancer growth [17, 18]. It has been proposed that androgens are able to counterbalance positive growth stimuli in the breast since they antagonize the estrogen-induced proliferative effects which are intimately linked to breast carcinogenesis [3]. In vivo studies in rhesus monkey evidenced that blocking the action of endogenous androgens results in a significant increase in mammary epithelial cell proliferation. Conversely, in ovariectomized animals, low doses of testosterone completely inhibit estrogen-induced mammary cell proliferation [19, 20].

In estrogen-receptor positive breast cancer cell lines the non-aromatisable androgen 5-α-dihydrotestosterone (DHT) inhibits both basal and estradiol-induced proliferation [7, 13]. Co-administration of testosterone suppresses the estradiol (E_2_)-mediated induction of MYC [21] and over-expression of the AR in MCF-7 cells markedly decreased estrogen receptor α (ERα) transcriptional activity [7, 14, 22] consistent with the notion that androgens inhibit estrogen-dependent signalling pathways.

Estrogens particularly exert their proliferative effects by induction of the G1 progression and G1/S transition through a mechanism involving the transcriptional control of cell cycle regulatory genes such as *CCND1,* encoding cyclin D1 [23, 24].

Overexpression of cyclin D1 is believed to endow mammary epithelial cells with a proliferative advantage by virtue of its contribution to pRB inactivation. Conversely, mice deficient in cyclin D1 activity show an autophagy-like process [25].

The correlation between *CCND1* expression levels and cellular proliferation in breast cancer cells has been also confirmed by *CCND1* silencing experiments, indicating cyclin D1 as a potential therapeutic target for breast cancer [26, 27].

We previously reported that *CCND1* represents a target gene of DHT-activated AR in MCF-7 breast cancer cells, evidencing the existence of a functional Androgen Response Element within the *CCND1* promoter, which mediates the DHT/AR inhibitory effects on basal breast cancer cell proliferation [13].

Since cyclin D1 has been shown to mediate E_2_-induced progression of MCF-7 from G1 into S phase, here we examined the possibility of the existence of an additional mechanism by which androgens, through their own receptor, may inhibit E_2_-induced cyclin D1 expression thus modulating estrogen-dependent breast cancer cell proliferation. In this report we demonstrate that in MCF-7 and in MCF-7 over-expressing the AR, DHT treatment decreases the E_2_-dependent expression of cyclin D1 protein as well as the transcriptional activity of the cyclin D1 gene promoter. We propose that the competition for the steroid receptor coactivator AIB1, that is important in the functional coupling of the ERα with the cyclin D1 promoter [28], may represent a possible mechanism through which AR can modulate ERα-mediated signalling pathway on cyclin D1 gene leading to the inhibition of breast cancer cell proliferation.

## Methods

### Reagents and antibodies

Dihydrotestosterone (DHT), hydroxyflutamide (OH-Fl) and estradiol (E_2_) were from Sigma Aldrich; antibodies against AR (441), cyclin D1 (M-20), ERα (F-10), GAPDH (FL-335), Actin (AC-15) were from Santa Cruz Biotechnology.

### Cell cultures

The human breast cancer MCF-7 (ATCC-HB-22) or human cervical cancer HeLa (ATCC- CRM-CCL-2) cell lines were acquired from ATCC (LCG Standards, UK). Cells were stored according to supplier’s instructions, and used within 6 months after frozen aliquots resuscitations. Cells were authenticated by short tandem repeat analysis (GenePrint® 10 System, Promega) at our Sequencing Core Facility. Mycoplasma negativity was tested monthly (MycoAlert, Lonza). Before each experiment, cells were synchronized in phenol red-free serum free media (PRF-SFM) for 24 h. All the experiments were performed in PRF-media containing 2.5% charcoal-treated (steroids depleted) Fetal Bovine Serum (PRF–CT). Cells were treated with 10^− 8^ M E2, and/or 10^− 7^ M DHT, and/or 10^− 6^ M OHFl.

### Cell proliferation assays

Cells were seeded on six-well plates (2x10^5^cells/well) in 2.5% PRF–CT. After 24 h, cells were exposed for 3 days to 10^-7^ M DHT and/or 10^−7^ M E_2_ and/or 10^− 6^ M OHFl, or left untreated (−) and then harvested by trypsin. Drug effects on cell proliferation were measured by counting cells using a Burker’s chamber; cell viability was determined by Trypan blue dye exclusion test as previously described [12].

### Plasmids, transfections and luciferase reporter assays

The following plasmids were used: Cyclin D1 promoter construct D1Δ-2966pXP2-Luc (a gift from Dr. A. Weisz, Università degli Studi di Salerno, Italy); wild-type AIB1 expression vector (a gift from Dr. B. O’Malley, Baylor College of Medicine, Houston TX USA); pcDNA3-AR (AR), encoding full-length androgen receptor, (a gift from Dr. M.J. McPhaul, UT-Southwestern Medical Center at Dallas TX, USA), the wild-type human ERα (HEGO) (a gift from Dr. P. Chambon, Université de Strasbourg, France).

Cells were transfected using Fugene 6 reagent (Roche Diagnostics) according to manufacturer’s instructions. *Renilla reniformis* luciferase expression vector pRL-Tk (Promega) was used to assess transfection efficiency. Luciferase activity was measured with the Dual Luciferase kit (Promega).

### Total RNA extraction, reverse transcription polymerase PCR and real-time RT-PCR assay

Total RNA was extracted from MCF-7 cells using TRIzol reagent and cDNA was synthesized by reverse transcription-polymerase chain reaction (PCR) method using a RETROscript kit. The expression of selected genes was quantified by real-time PCR using iCycler iQ Detection System (Bio-Rad, Hercules, CA) as previously described [29]. Five microliters of diluted (1:3) cDNA was analyzed using SYBR Green Universal PCR Master Mix, following the manufacturer’s recommendations. The primers (Invitrogen) for Cylin D1 gene were: *forward*: 5′-CCGTCCATGCGGAAGATC-3′; reverse: 5′-AAACGTGGGTCTGGGCAA-3′. Each sample was normalized on the basis of its 18S ribosomal RNA content. The 18S quantification was performed using a TaqMan Ribosomal RNA Reagent kit (Applied Biosystems) following the method provided in the TaqMan Ribosomal RNA Control Reagent kit. The relative gene expression levels were normalized to a calibrator that was chosen to be the basal, untreated sample. Final results were expressed as n-fold differences in gene expression relative to 18S ribosomal RNA and calibrator, calculated following the ΔΔThreshold cycle (Ct) method, as published previously. Assays were performed in triplicate.

### Immunoprecipitation and WB

Total cell proteins were obtained from 70% confluent cell cultures. Immunoprecipitation (IP) and Western blotting (WB) were performed as previously described [22, 30]. The images were acquired by using an Epson Perfection scanner (Epson, Japan) using Photoshop software (Adobe). The optical densities of the spots were analyzed by using ImageJ software (NIH; http://rsb.info.nih.gov/IJ).

### Chromatin Immunoprecipitation (ChIP) assay and real time ChIP

ChIP assay was performed as previously described [29]. Immuno-cleared chromatin was precipitated with anti-AIB1 or anti-ERα antibody. Immunoprecipitated DNA was analyzed in triplicates by real-time PCR by using 5 μl of the diluted (1:3) template DNA. The following primers (Invitrogen) spanning the AP-1 site of the Cyclin D1 promoter were used: *forward* 5′- CTTCGGTGGTCTTGTCCCA- 3′ and reverse 5′- CTTCCCGTGCCGGCAATTTA- 3′.

Real-time PCR data were normalized with respect to unprocessed lysates (input DNA). Inputs DNA quantification was performed by using 5 μl of the diluted (1/50) template DNA. The relative antibody bound fractions were normalized to a calibrator that was chosen to be the basal, untreated sample. Final results were expressed as percent to the relative inputs as previously described.

### RNA silencing

AIB1 silencing experiments were performed using Stealth™ Select RNAi (Invitrogen) annealed duplexes. Non-specific (NS) siRNA was used as a control for non-sequence-specific effects. Cells were transfected with 100 pmol of siRNA AIB1 or NS siRNA, using Lipofectamine 2000 (Invitrogen Life Technologies), following manufacturer’s instructions.

### Statistical analysis

Statistical analysis was performed using ANOVA followed by Newman-Keuls’ testing to determine differences in means. All data are reported as the mean ± SD of three different experiments, each performed in triplicates. * *p* ≤ 0.05 vs control.

## Results

### Inhibition of estrogen-dependent proliferation by androgen receptor over-expression

We previously demonstrated that MCF-7 cells are androgen-responsive and that DHT treatment induces a transient increase in AR protein levels [22] similar to that seen in other cell types [31, 32].

**Fig. 1:**
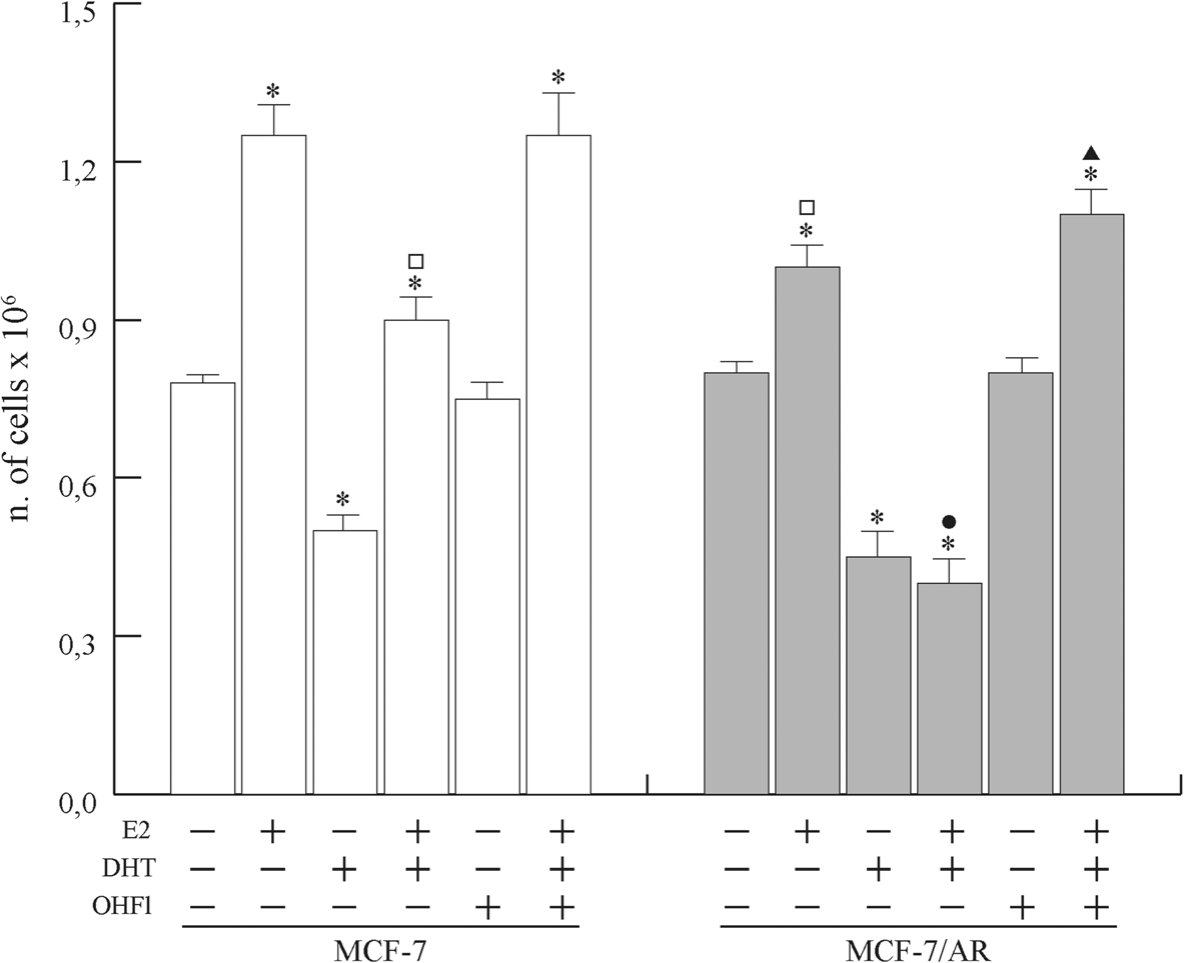
Over-expressed androgen receptor inhibits E_2_-dependent MCF-7 cells proliferation. MCF-7 cells and and MCF-7 transiently over-expressing AR (MCF-7/AR), were synchronized in PRF and treated with 10^− 7^ M E_2_, and/or 10^− 7^ M DHT, and/or 10^− 6^ M OH-Fl in steroids depleted PRF-CT for 3 days. Data represent a mean ± s.d. of three independent experiments, each in duplicate. *p ≤ 0.05 vs untreated MCF-7 cells; ^□^*p* ≤ 0.05 vs. E_2_-treated MCF-7 cells; ^●^*p* ≤ 0.05 vs E_2_ + DHT treated MCF-7 cells; ^▲^*p* ≤ 0.05 vs E_2_ + DHT treated MCF-7/AR

Thus, we investigated the role of DHT-dependent signalling on the E_2_-induced proliferation of the ERα-positive MCF-7 cells. Consistent with previous reports [13, 14], prolonged DHT administration resulted in a significant reduction of basal as well as E_2_-dependent MCF-7 cell proliferation (Fig. 1). To better investigate the role of androgen receptor, MCF-7 cells were transiently transfected with the pcDNA3-AR expressing the full length AR (MCF-7 cells/AR). The ectopic overexpression of AR “per se” reduced E_2_-dependent cell proliferation and further potentiates the inhibitory effects determined by DHT administration. Addition of the androgen antagonist hydroxyflutamide (OHFl) effectively reversed the inhibition of E_2_-induced cell growth exerted by DHT, suggesting that the effect was mediated by AR.

### Inhibition of E_2_-induced cyclin D1 gene expression and promoter activity by androgen receptor over-expression

Since a key rate-limiting event in mitogenic estradiol signalling leading to S-phase entry is the induction of cyclin D1 [33] we investigated whether AR activation and/or its over-expression might modulate cyclin D1 expression.

**Fig. 2:**
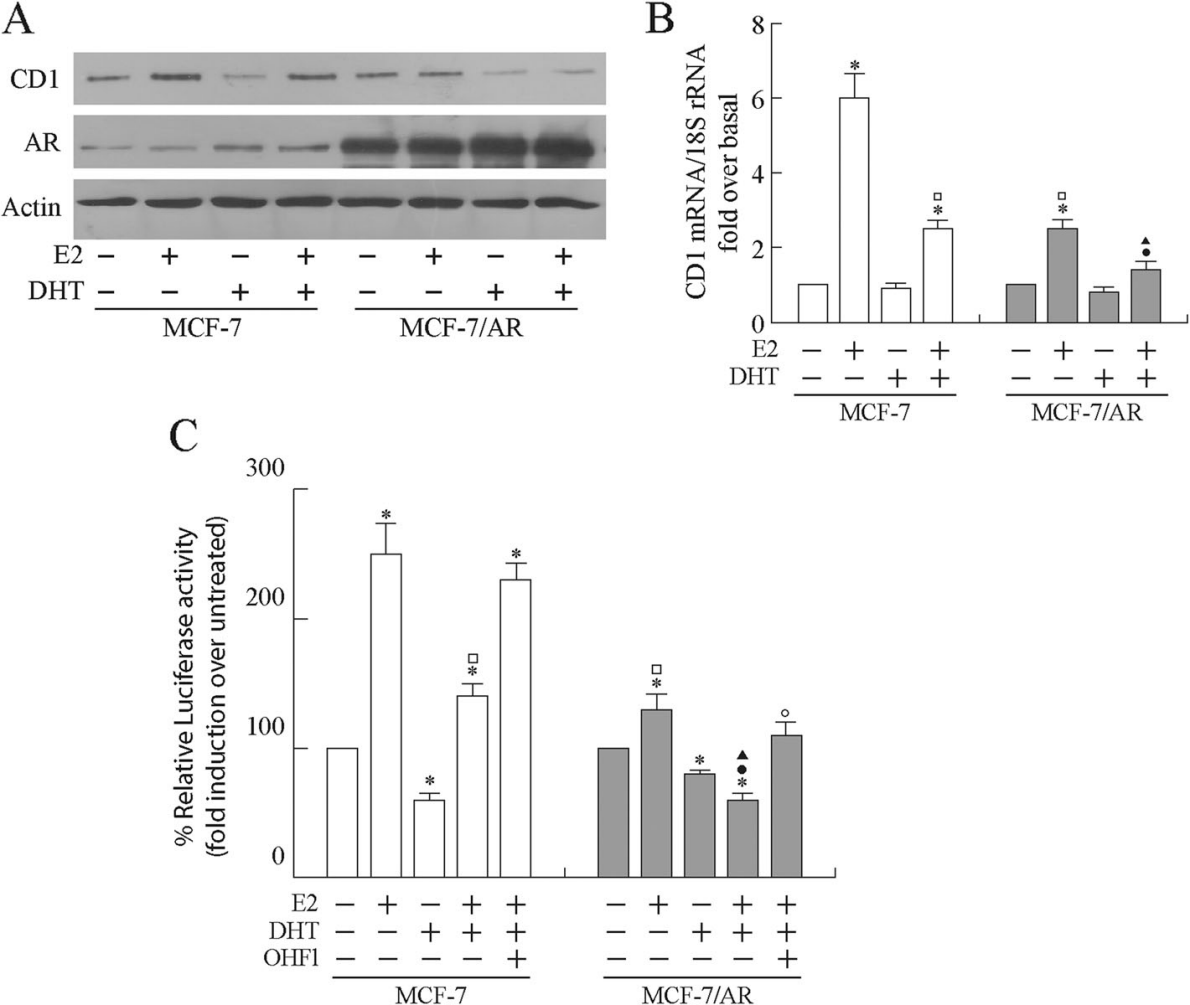
Estrogen induction of cyclin D1 expression and promoter activity is reduced by over- expression of androgen receptor. **a** Western blotting analysis of Cyclin D1 (CD1). MCF-7 and MCF-7/AR cells were treated as indicated. Actin was assessed as control of protein loading. **b** Quantitative Real Time RT–PCR from MCF-7 and MCF-7/AR cells treated as indicated. 18S rRNA was determined as control. Columns are the mean of three independent experiments each in triplicate; bars, SD; *p ≤ 0.05 vs untreated MCF-7 cells; ^□^*p* ≤ 0.05 vs. E_2_-treated MCF-7 cells; ^●^*p* ≤ 0.05 vs E_2_ + DHT treated MCF-7 cells. **c** MCF-7 and MCF-7/AR cells were transiently transfected with pCD1prom-Luc and treated as indicated. Columns are mean of three independent experiments and expressed as fold induction over untreated, which was assumed to be 100%; bars SD; *p ≤ 0.05 vs untreated MCF-7 cells; ^□^*p* ≤ 0.05 vs. E_2_-treated MCF-7 cells; ^●^*p* ≤ 0.05 vs E_2_ + DHT treated MCF-7 cells; ^▲^*p* ≤ 0.05 vs. E_2_-treated MCF-7/AR cells; ^○^*p* ≤ 0.05 vs. E_2_ + DHT treated MCF-7/AR cells

To this aim, MCF-7 cells were transiently transfected with an empty vector or with a full length AR expression plasmid and left untreated or treated with E_2_ and/or DHT for 48 h. A significant reduction in the E_2_-induced cyclin D1 protein expression levels was observed following DHT co-treatment in MCF-7 cells. Interestingly, AR overexpression per se determined a decrease of cyclin D1 protein content in response to E_2_ stimulation, which was further reduced following DHT coadministration (Fig. 2a). A similar regulatory pattern was observed in terms of mRNA expression levels (Fig. 2b).

Next, we examined the possibility that AR activation by its own ligand and/or AR over-expression might negatively modulate the E_2_/ERα-induced *cyclin D1* promoter transcriptional activity.

As shown in Fig. 2c, in MCF-7 cells, a cyclin D1 promoter construct driving luciferase expression was induced by E_2_ but significantly inhibited following DHT co-administration. The overexpression of AR resulted in the complete loss of the transcriptional signal induced by E_2_ when compared with hormone stimulated activity in the absence of exogenous AR. Additionally, in these experimental conditions, a further decrease in E_2_-dependent cyclin D1 promoter activity was observed following DHT treatment. The androgen-dependent inhibition of E_2_-activated signalling on cyclin D1 gene promoter was abrogated by the addition of the androgen antagonist OHFl, confirming the involvement of AR.

### AIB1 overexpression rescues AR repression of estradiol-induced transcriptional activity of cyclin D1 promoter

The capacity of AR to compromise the transcriptional response dependent on a second receptor such as ERα, implies that shared components of the transcriptional machinery are involved [22, 34]. Therefore AR and ERα might use a common pool of co-factors present in limiting cellular concentrations. In this concern, we investigated the role of the steroid receptor coactivator AIB1, that is important in the functional coupling of ERα with the cyclin D1 promoter [28, 35].

**Fig. 3:**
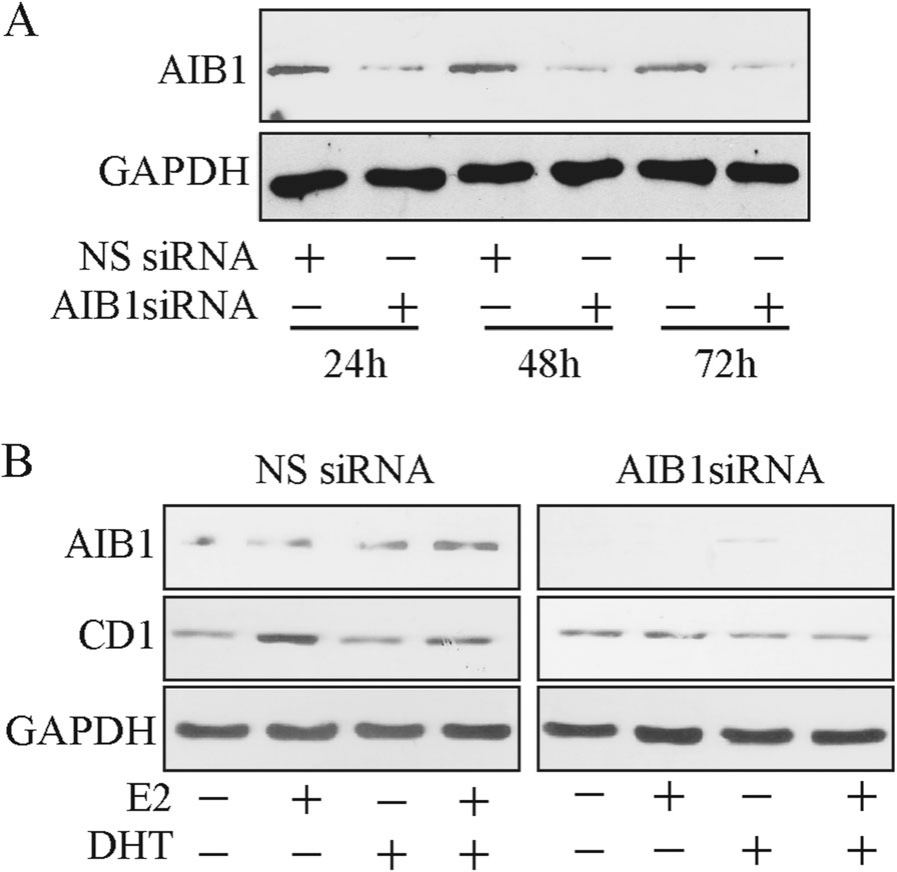
AIB1 is crucial for E_2_-induced Cyclin D1 expression (**a**) Western blotting analysis of AIB1. MCF-7 cells were transfected with non specific (NS) or targeted against AIB1 siRNA at different times, as indicated. GAPDH was used as loading control. **b** Western blotting analysis of AIB1 and CD1. MCF-7 cells were transfected with non specific (NS) or targeted against AIB1 siRNA and treated as indicated. GAPDH was used as loading control. Results are representative of three independent experiments

To this aim, we first used a AIB1 siRNA approach, to selectively reduce AIB1 expression in MCF-7 cells. The AIB1 siRNA produced a > 80% reduction in cellular AIB1 protein levels, which were still repressed after 72 h (Fig. 3a). As shown in Fig. 3b treatment with AIB1 siRNA completely negated the increase in cyclin D1 protein expression induced by estradiol.

Interestingly, this pattern of cyclin D1 expression is similar to the one observed following AR over-expression (Fig. 2b), supporting the hypothesis that AIB1 is essential for E_2_-dependent cyclin D1 expression.

Thus to assess whether AIB1 squelching might be involved in the transcriptional interference of AR on ERα transcriptional signal, we tested whether AIB1 overexpression could rescue AR repression of estradiol-induced transcriptional activity on cyclin D1 promoter.

**Fig. 4:**
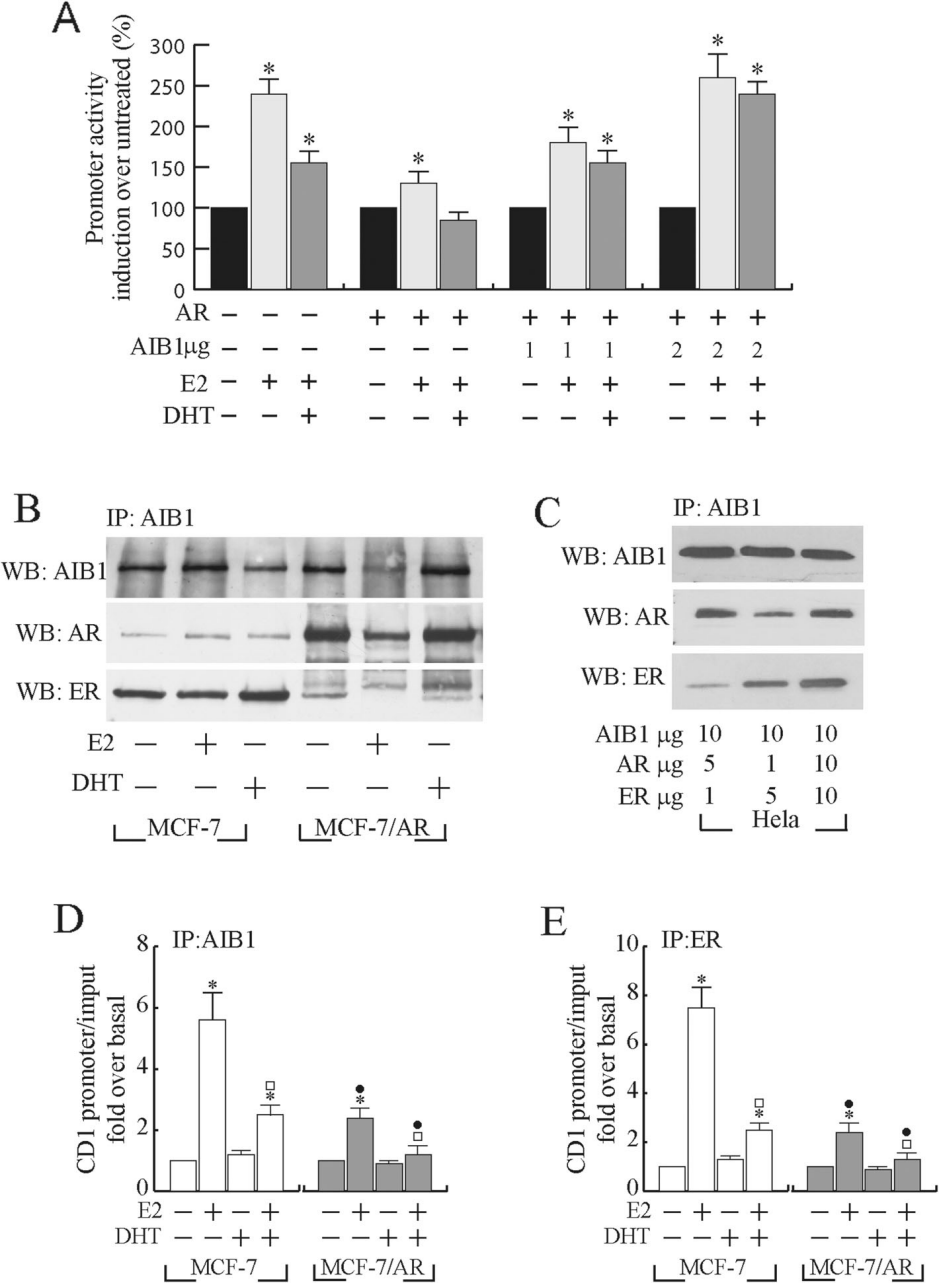
Over-expressed androgen receptor competes with ERα for AIB1 interaction. **a** MCF-7 cells, were transiently co-transfected with pCD1prom-Luc (0.25 μg /well) and /or pcDNA3-AR (AR) and/or increasing amounts (given in μg/well) of full-length AIB1 expression plasmid (AIB1), and treated as indicated. Columns are mean of three independent experiments and expressed as fold induction over untreated, which was assumed to be 100%; bars SD; *p ≤ 0.05. **b** Total cell extracts from MCF-7 and MCF-7/AR were immunoprecipitated (IP) with an anti-AIB1 antibody and immunoblotted (WB) to detect AIB1, ER and AR protein levels. Results are representative of three independent experiments. **c** Total cell extracts from Hela cells transiently cotransfected with different amounts (given in μg/dish) of pcDNA3-AR (AR), Hego (ER) or full-length AIB1 expression plasmid as indicated were immunoprecipitated (IP) with an anti-AIB1 antibody and immunoblotted (WB) to detect AIB1, ER and AR protein levels. Results are representative of three independent experiments. **d** and **e** ChIP-qPCR performed on MCF-7 cells and MCF-7/AR cells using anti-AIB1 (**d**) or anti- ERα (**e**) antibodies, as indicated. IgG was used as control. Columns are the mean of three independent experiments. Bars, SD; *p ≤ 0.05 vs untreated MCF-7 cells; ^*□*^*p* ≤ 0.05 vs. E_2_-treated MCF-7 cells; ^●^*p* ≤ 0.05 vs E_2_ + DHT treated MCF-7 cells

As shown in Fig. 4a, progressively increasing amounts of ectopic AIB1 were able to restore the E_2_-dependent activation of cyclin D1 promoter activity, although in the presence of exogenous AR expression. Thus, AIB1 over-expression is able to completely abrogate the inhibitory effect induced by overexpressed AR and to re-establish E_2_-induced activity of cyclin D1 promoter in MCF-7 cells.

### AIB1 interaction with either AR or ERα is related to the intracellular content of both steroid receptors

As AIB1 has been reported to be capable to bind and coactivate both ERα and AR [36, 37] we investigated if AIB1 interaction with ERα and/or AR might be influenced by the intracellular levels of the two steroid receptors. To this aim, AIB1/ERα and/or AIB1/AR complex formation was analysed by co-immunoprecipitation assay in both MCF-7 and MCF-7/AR cells (Fig. 4b). Interestingly, in MCF-7 cells, which express high levels of endogenous ERα and low levels of AR [22], AIB1 co-immunoprecipitates predominantly with ERα in all the examined experimental conditions. On the contrary, following AR overexpression, a prevalent interaction of AIB1 with AR was observed. Specifically, in MCF-7/AR overexpressing cells we found a substantial decrease of AIB1/ERα complex and a concomitant increase of AIB1/AR interaction, compared to MCF-7 cells. As depicted in Fig. 4c similar experimental conditions reproduced in Hela cells co-expressing ectopic AIB1, ERα and AR, determined analogous results. Indeed, to further prove whether AIB1 is capable to interact with AR and/or ERα in relationship to their intracellular levels, we next performed studies with HeLa cells, a well-known experimental model, which do not express AR or ERα [38, 39]. In these experiments, coimmunoprecipitation assays were carried out in HeLa cells transiently cotransfected with both ERα and AR in a ratio of ERα /AR = 1:5 or at a ratio of ERα /AR = 5:1 in the presence of an excess of AIB1. Again, when AR content is higher than ERα (ratio ERα/AR = 1:5), AIB1 coimmunoprecipitates mainly with AR while, in the presence of an excess of ERα (ratio ERα /AR = 5:1), AIB1 primarily coimmunoprecipitates with ERα (Fig. 4c).

It has been reported that AIB1 is required for ERα recruitment onto the estrogen responsive region of the cyclin D1 promoter [28, 40]. Thus, to highlight the biological implication of AR/ERα competition for a shared coactivator such as AIB1 in the regulation of cyclin D1 promoter, we evaluated its recruitment on cyclin D1 promoter by chromatin immunoprecipitation (ChIP) assay in MCF-7 and MCF-7/AR cells (Fig. 4d). Protein-chromatin complexes were immunoprecipitated using specific antibodies against AIB1 or ERα. The presence of the specific promoter sequence in the chromatin immunoprecipitates was analyzed by Real-time PCR using specific primers spanning the estrogen-responsive region of the cyclin D1 promoter that contains an AP-1 binding site.

As indicated in Fig. 4d, in MCF-7 cells E_2_-induced recruitment of AIB1 on cyclin D1 proximal promoter was decreased by DHT co-administration. Alongside, in the same experimental conditions, also E2-dependent ERα binding to the AP-1 containing region was reduced (Fig. 4e). Interestingly and consistent with the above reported data, AR overexpression greatly counteracts either the AIB1 or ERα occupancy of cyclin D1 promoter induced by E_2_.

## Discussion

Estrogens play a central role in the proliferation and the differentiation of normal mammary epithelial cells as well as the development and progression of breast cancer [41–43]. Indeed, human breast tumorigenesis is promoted by enhanced activity of the estrogen receptor α (ERα) that regulates the transcription of target genes, which in turn direct cellular proliferation [44]. Among these genes, *cyclin D1* plays a pivotal role, as highlighted by several lines of evidences. In cyclin D1 knockout mice, mammary gland development is profoundly impaired and more evident during pregnancy when ovarian steroids fail to induce their massive proliferative changes [45, 46]. Cyclin D1 over-expression has been reported in about 50% of invasive breast cancer [47] and strongly correlates with ER levels [48–50].

The mechanism by which estrogens regulate cyclin D1 levels in hormone-responsive breast cancer cells is mainly transcriptional. Although no estrogen-responsive element- (ERE) –related sequence has been identified in the cyclin D1 promoter, several potential estrogen-responsive sites have been mapped in the cyclin D1 proximal promoter [24, 40, 51–53].

In the present study we provide a mechanism by which ligand-activated AR, down-regulates estrogen-dependent MCF-7 human breast cancer cell proliferation by inhibiting the ability of E_2_/ERα signalling to direct transcription of the cyclin D1 gene promoter. Indeed, in this cell type, co-administration of the non aromatisable androgen DHT, down-regulates the estrogen-dependent induction of cyclin D1 expression at both mRNA and protein levels as well as of its gene promoter activity.

It is recognized that ERα-mediated transcription is a highly complex process involving a multitude of coregulatory factors and cross-talk among distinct signalling pathways [22, 54]. A number of non-mutually exclusive mechanisms by which the action of steroid receptors might be competitive do exist, including homo- and heterodimers formation, structural analogy of activating ligands, binding to shared DNA response elements or sequestration of transcriptional co-regulators present in limiting cellular concentrations [7, 22, 55, 56]. Here, we demonstrated that the negative interference of ligand-activated and/or over-expressed AR on E_2_/ERα dependent transcriptional induction of cyclin D1 involves sharing of the steroid receptor coactivator AIB1, whose abnormal expression is associated with malignancies in estrogen target tissues. Indeed AIB1 was originally identified on the basis of its frequent amplification and over-expression in ovarian and breast cancers [57, 58]. In addition, AIB1 amplification correlates in primary breast cancers with ERα positivity and tumor size [59, 60] and, very recently, it has been proposed that, in ERα-positive/HER2-negative invasive breast carcinoma, AIB1 could serve as a new putative prognostic biomarker, with its expression (high AIB1 vs low AIB1) being associated to breast cancer mortality [61]. More, interestingly, AIB1 has an unique role in regulating estrogen-dependent signalling as it is essential for ERα transcriptional activity [62, 63]. This peculiarity of AIB1 serves as a mechanism by which it influences the growth of hormone-dependent breast cancer as suggested by the observation that depletion of AIB1 affects estrogen-dependent cell proliferation and survival in ER-positive MCF-7 human breast cancer cells, causing a reduction of MCF-7 xenografts growth in mice [64, 65]. Specifically, in MCF-7 cells, AIB1 represents a rate-limiting factor for estrogen-dependent growth [64] since its cellular levels influence the ability of ERα to interact with the cyclin D1 promoter in an estrogen-dependent manner [28].

Our data evidence that, in MCF-7 cells, exogenously expressed AIB1 reverses the AR repression of E_2_-dependent transcriptional activity of cyclin D1 promoter suggesting that the transcriptional interference between AR and ERα on cyclin D1 promoter might actually involve competition for limiting amounts of AIB1 in the cell. It has been proposed that coactivators preferentially interact with receptors depending on cell type, ligand and promoter context, which could contribute to the specificity of the physiological response [62, 66]. In our experimental models, the ability of AIB1 to modulate AR/ERα interplay is dependent on the steroid receptor cellular content since in MCF-7 cells expressing high levels of endogenous ERα and low levels of AR, AIB1 interacts predominantly with ERα. In contrast, AR over-expression induces a dominant interaction of AIB1 with AR. This coactivator squelching between the two steroid receptors impacts on ERα-driven transcription of growth regulatory genes. In MCF-7 cells, specific AR ligand-activation, which is associated with an increased AR cellular content [14, 22], is able to determine a significant decrease in the estrogen-induced recruitment of AIB1 onto the AP-1 site containing region of the cyclin D1 promoter. Consistent with the notion that AIB1 is fundamental for ERα recruitment within the estrogen-responsive sequence of the cyclin D1 promoter [28], a similar reduction in ERα occupancy of the AP-1 site containing region was also evidenced**.** Our results well correlate with previous findings showing that loss of AIB1 affects ERα-mediated signalling by both directly inhibiting transcriptional initiation and blocking ERα turnover, which may further compromise transcriptional regulation by the receptor [63]. Besides we showed that specific AIB1 knock-down completely abrogated E_2_ effect on cyclin D1 expression.

In conclusion, we demonstrated that the physical squelching of the AR/ER shared coactivator AIB1 may represent at least one of the several potential mechanisms through which AR can negatively modulate ERα-mediated signalling pathway and inhibit breast cancer cells proliferation. More precisely, our study emphasizes how coactivator availability may be indeed crucial in determining the transcriptional profile of the cells. Although in a simplified experimental model like ours, findings from the present study reinforce previous observations demonstrating, in ERα-positive breast cancer cells, a reciprocal interference between DHT- and estradiol-induced transcriptional program [67], able to shape a unique transcriptional network. We cannot exclude that other receptors could also contribute/influence the interplay between AR and ERα signaling [68, 69]. Indeed, it is well established that the response of cells to circulating steroid hormones is not the sum of individual hormone action but the result of a functional interaction between different nuclear receptors activating many downstream effector genes and pathways [70, 71]. In this contest it is relevant the finding that even the progesterone receptor (PR) has been demonstrated to be able to modulate ERα-DNA binding, directly reprogramming ERα-dependent transcriptional programs within breast cancer cells and inhibiting estrogen-mediated growth of ERα-positive cell line xenografts [72]. Identification of the potential mechanisms underlying the functional cross-talk between different steroid receptors and the involved specific genes and pathways could provide new hints in hormone receptor actions in breast cancer.

## Conclusions

In summary, our study underlines, once more, the existence in breast cancer cells of a dynamic interplay between AR and ERα signalling pathways that strictly depends on the hormonal cellular milieu. The biological significance of the AR-induced inhibition of cyclin D1 expression is highlighted by clinical studies in ERα-positive breast cancers, showing a better response to adjuvant therapy in cancer patients with cyclin D1 low/moderate expression than those with high expression of cyclin D1 [73, 74]. In this context, the expression and the functional activity of the AR in breast tissues and tumors, by opposing the estrogen signalling, might play a critical role in regulating cellular proliferation and tissue homeostasis.

Given the high frequency of AR expression in the majority of ERα-positive breast tumors, the AR/ERα crosstalk supports the intriguing idea of coupling androgen-based therapy with therapies targeting other important pathways, for the treatment of ERα-positive breast cancer patients.

## Data Availability

Data produced in this study are available from the corresponding author on reasonable request.

## Abbreviations

AIB1: Amplified in Breast Cancer 1
AR: Androgen receptor
DHT: 5-α-dihydrotestosterone
E_2_: estradiol
ERα: Estrogen Receptor alpha
